# Impact of Fasting Lipid Profile on Chronic Kidney Disease Patients Having Fatty Liver Disease

**DOI:** 10.7759/cureus.11146

**Published:** 2020-10-25

**Authors:** Muhammad Sohaib Asghar, Maira Hassan, Uzma Rasheed, Syed Jawad Haider Kazmi, Noman A Khan, Faran Khalid, Ayesha Anum, Saira Anwar

**Affiliations:** 1 Internal Medicine, Dow International Medical College, Dow University Hospital, Dow University of Health Sciences, Karachi, PAK; 2 Internal Medicine, Liaquat National Hospital, Karachi, PAK; 3 Emergency Medicine, Liaquat National Hospital, Karachi, PAK; 4 General Surgery, Liaquat National Hospital, Karachi, PAK

**Keywords:** kidney, dialysis, fatty liver, nafld, lipid, ckd

## Abstract

Background and objectives

Chronic kidney disease (CKD) share a common pathophysiology with non-alcoholic fatty liver disease (NAFLD). This study aims to identify the lipid derangements in patients of CKD and to associate them with radiological evidence of NAFLD.

Material and methods

A cross-sectional observational study was performed in a tertiary care hospital, to include all chronic kidney disease patients (n=238) through non-probability consecutive sampling. The criteria for inclusion were baseline estimated Glomerular filtration rate (eGFR) below 60 ml/min/1.73m^2^ for at least three months and chronic renal parenchymal changes on ultrasound. Two study groups were identified based on ongoing hemodialysis, while two further study groups were identified based on radiological evidence of fatty liver disease.

Results

The mean age of the study population was 48.52 ± 9.44 years with no difference amongst hemodialysis status, females elder than males (p= 0.027), those with fatty liver were much younger (p=0.014), and the most common age group below 50 years (p=0.005) among the fatty liver group. Radiological evidence of NAFLD was found amongst two-third of the study group with the status of hemodialysis indifferent among the study population (p=0.436). The mean values amongst fatty liver versus non-fatty liver groups revealed high creatinine, alanine transaminase (ALT), high-density lipoprotein (HDL), triglycerides (TG), and very-low-density lipoprotein (VLDL) in the fatty liver group, low-density lipoprotein (LDL) and total cholesterol (TC) were indifferent amongst the groups, while LDL/HDL ratio was higher in the non-fatty liver group.

Conclusion

A significantly higher HDL was found in fatty liver associated with CKD as compared to the non-fatty liver group.

## Introduction

Chronic kidney disease (CKD) affects 8% of the total world population, with its prevalence increase with increasing age. CKD and Non-alcoholic fatty liver disease (NAFLD) share a common pathophysiology. Like NAFLD, CKD is characterized by a deranged cellular substrate metabolism, fat deposition, which triggers oxidative stress and inflammatory and pro-fibrotic responses to drive the progression of both disease processes [1]. Hepatic steatosis with CKD results in a decrease in eGFR (estimated GFR) which may increase the severity of CKD [2]. CKD also cause dyslipidemia, and hyperlipidemia has proven to increase the disease progress, the most common lipid abnormalities which have been noted are a decrease in High-density lipoprotein (HDL) and hypertriglyceridemia [3]. CKD is also accompanied by altered lipid metabolism as a consequence of nephrotic syndrome and causes a change in apolipoprotein profile and plasma lipid level [4]. In renal transplant recipients Total Cholesterol (TC), Very low-density lipoprotein (VLDL), Low-density lipoprotein (LDL), and triglycerides are high, whereas HDL is reduced [5]. As CKD and NAFLD show common pathogenesis, they have potentially the same therapeutic targets. Most of them are being evaluated in phase II randomized control trials, and some of them, such as peroxisome proliferator-activated receptor (PPAR-α/δ) agonists, Farnesoid X receptor (FXR) agonists, and incretin analogues are showing good results [1].

Fatty liver disease is a common term for people suffering from non-alcoholic fatty liver disease (NAFLD), which is defined as the presence of fat in the liver tissue [6], seen either by imaging or by histology in the absence of any history of significant alcohol consumption, use of steatogenic medicines or related hereditary disorders [7]. A more severe subtype of NAFLD is non-alcoholic steatohepatitis (NASH), where there is associated inflammation and scarring of the liver tissue alongside fat accumulation and is a common indicator for a liver transplant [8]. NAFLD is the most typical disease of liver worldwide having a global prevalence of 25.24% [9], with the highest in the Middle East (31.8%) and South America [10]. Non-alcoholic fatty liver disease is associated with other metabolic co-morbidities, including obesity, type-II diabetes mellitus, hypertension, and hypocholesterolemia.

Lifestyle modification that includes increased physical activity and low-calorie diets can reduce high levels of liver enzymes [11]. Weight reduction is one of the important factors in the treatment of NAFLD. It is recommended an overall 7-10% of total weight loss causes marked reduction in hepatic steatosis [12]. Therefore, a moderate-intensity workout weekly can be used to improve the outcome of the disease. There is no one dietary regime that can treat non-alcoholic fatty liver (NAFL). However, it is seen that dietary changes involving low carbohydrate diet and increased fibre intake, mostly consuming green vegetables and fresh fruits has shown positive effects towards insulin sensitivity and on cholesterol levels of the patients [13].

Pharmacotherapy used for the treatment of NAFLD includes those directed at improving insulin resistance, reducing oxidative stress, decreasing hepatic fibrosis, improving the underlying metabolic syndrome, or promoting weight loss. Oxidative stress and increased production of inflammatory cytokines are the two leading molecular causes of the development of NAFLD [14]. Hence, Vitamin E, a potent antioxidant is the acknowledged pharmacological treatment of NAFLD as it reduces the oxidative stress in the cells. Vitamin E therapy has shown notable recovery in inflammation, ballooning, and resolution of steatohepatitis in patients with advanced NASH in adults [10], and children [15].

Statins, on the other hand, are safe agents for the treatment of hyperlipidemia, widely used for the treatment of the metabolic syndrome. They inhibit β-Hydroxy β-methylglutaryl-CoA (HMG-CoA) reductase enzyme in cholesterol synthesis, reducing the circulating LDL cholesterol; they also have antioxidant, anti-inflammatory, and immunomodulatory properties [16]. In the treatment of NAFLD, statins in patients with a body mass index of more than 27.5 lower the prevalence of steatosis [12], and has shown a reduction in oxidized low-density lipoprotein in patients with advanced fatty liver disease like NASH [17]. Furthermore, treatment with statins has also shown improvement in the grade of inflammation and reduced overall rate of fibrosis in patients with NAFLD [18].

This study aims to identify the lipid derangements occurring in patients of chronic kidney disease and to associate them with radiological evidence of non-alcoholic fatty liver disease. The secondary aim is to find out whether hemodialysis has any effect on the derangements of lipid markers.

## Materials and methods

A cross-sectional observational study was conducted in the nephrology department of a tertiary care hospital having the largest dialysis unit in the city. A sample size of 218 was calculated by using a Rao-soft sample size calculator (http://www.raosoft.com/samplesize.html), with a population size of 500, a response distribution of 50%, and a confidence interval of 95%. Informed consent was taken from all the participants of the study.

A Proforma was designed to have two sections; the first section covered demographic details including name (as optional), age (in years), gender, co-morbidities including hypertension, diabetes, and others, the status of hemodialysis, and radiological findings of abdominal ultrasound. While the second section includes the laboratory markers (including fasting lipid profile). The study excluded all the patients having diagnosed with viral hepatitis, hepatocellular carcinoma, alcohol use, or any other liver morbidity (except NAFLD leading to CLD). The study participants were included via a non-probability consecutive sampling technique. The criteria for inclusion were baseline eGFR (estimated GFR) below 60 ml/min/1.73m^2^ for at least three months and chronic renal parenchymal changes on ultrasound. Clean voided, midstream urine samples were collected from all the subjects and processed to document proteinuria and abnormal urinary sediments. Two study groups were identified on the basis of ongoing hemodialysis, while two further study groups were identified on the basis of radiological evidence of fatty liver disease.

After the data collected, it was analyzed on IBM-SPSS version 25.0 (Armonk, NY, USA), and results were obtained. The statistical difference was calculated via independent sample t-test, chi-square, and Fisher’s exact test, and considered significant if <0.05. A receiver operating characteristic curves were obtained to discrete the lipid markers association with fatty liver disease. Laboratory values were also correlated using both Pearson’s and spearman’s correlation appropriately.

## Results

A total of 238 CKD patients met the inclusion criteria with a mean age of 48.52 ± 9.44 years with no difference amongst hemodialysis, females elder than males (p=0.027), and those with fatty liver were much younger (p=0.014). The most common age group has been below 50 years (p=0.005) among the fatty liver group. Two-third of the study population were comprised of males amongst whom three-fourth had radiological evidence of fatty liver (p=0.002). The most common co-morbidities (other than CKD) amongst the study groups were diabetes (p<0.001), hypertension (p=0.040), CLD (p=0.002) among others with many of them were statistically more common in the fatty liver group. The descriptive statistics of the study population are stated in Table 1.

**Table 1:** Demographic data of the study population. * indicates independent sample t-test. ** indicates chi-square test. † indicates Fisher’s Exact test. Abbreviations: DM, diabetes mellitus; HTN, hypertension; IHD, ischemic heart disease; CVA, cerebrovascular accident; HIV, human immunodeficiency virus; CKD, chronic kidney disease; T.B, tuberculosis; CLD, chronic liver disease; NAFLD, non-alcoholic fatty liver disease.

Demographic data of the study population (n=238)						p-value
1	Age (in years)	Age Group	<50		>50	-
		Total	153 (64.3%)		85 (35.7%)	
		Hemodialysis	72 (52.9%)		40 (47.1%)	1.000**
		No Hemodialysis	81 (52.9%)		45 (47.1%)	
		Fatty liver	112 (70.4%)		47 (29.6%)	0.005**
		No fatty liver	41 (51.9%)		38 (48.1%)	
2	Mean age (in years)	48.52 ± 9.44				-
		Males: 47.38 ± 8.11		Females: 50.38 ± 11.09		0.027*
		Hemodialysis: 48.58 ± 8.05		No Hemodialysis: 48.46 ± 10.55		0.917*
		Fatty liver: 47.52 ± 9.82		No Fatty liver: 50.53 ± 8.32		0.014*
3	Gender	Males: n=148 (62.2%)		Females: n=90 (37.8%)		-
		Hemodialysis: n=68 (45.9%)		Hemodialysis: n=44 (48.9%)		0.659**
		No Hemodialysis: n=80 (54.1%)		No Hemodialysis: n=46 (51.1%)		
		Fatty liver: n=110 (74.3%)		Fatty liver: n=49 (54.4%)		0.002**
		No fatty liver: n=38 (25.7%)		No fatty liver: n=41 (45.6%)		
4	Comorbidities (other than CKD)	Frequency of Diseases	Fatty Liver (n=159)		No fatty liver (n=79)	-
		DM: 57.56% (n=137)	69.8% (n=111)		25.7% (n=26)	<0.001**
		HTN: 27.73% (n=66)	68.2% (n=45)		31.0% (n=21)	0.040**
		IHD: 13.86% (n=33)	9.7% (n=19)		13.8% (n=14)	0.999**
		CVA: 6.30% (n=15)	3.2% (n=8)		6.9% (n=7)	0.732**
		Autoimmune: 7.98% (n=19)	2.2% (n=7)		13.8% (n=12)	0.057**
		Hypothyroidism: 6.72% (n=16)	4.3% (n=8)		17.2% (n=8)	0.526**
		HIV: 2.94% (n=7)	3.2% (n=4)		0.0% (n=3)	1.000^†^
		T.B: 1.26% (n=3)	1.1% (n=1)		3.4% (n=2)	0.576^†^
		CLD: 11.34% (n=27)	61.3% (n=23)		69.0% (n=4)	0.002**
		No co-morbidities (other than CKD): 21.42% (n=51)	22.6% (n=21)		20.7% (n=30)	0.008**
5	Evidence of NAFLD (on ultrasonography)	Present: n=159 (66.8%)		Absent: n=79 (33.2%)		0.436**
6	Status of hemodialysis	Yes: n=112 (47.1%)		No: n=126 (52.9%)		

Radiological evidence of NAFLD was found amongst two-third of the study group. While the status of ongoing hemodialysis was indifferent among the study groups (p=0.436). A comparative analysis was done amongst the group with hemodialysis as compared to those not on hemodialysis shown indifferent mean values of all the lipid markers. The only statistical difference found was increased creatinine in the hemodialysis group (p= 0.004), as shown in Table 2.

**Table 2:** Comparison of lipid and biochemical markers amongst the status of hemodialysis. P-Value calculated by independent sample t-test (* denotes significant values). Abbreviations: HDL, high-density lipoprotein; LDL, low-density lipoprotein.

#	Laboratory investigations	All patients (n=238)	Grouping variables		p-value
			On Hemodialysis (n=112)	No Hemodialysis (n=126)	
1	Serum Creatinine (mg/dl)	5.12 ± 3.38	5.81 ± 4.37	4.51 ± 1.99	0.004*
2	Serum Albumin (g/dl)	3.28 ± 0.57	3.32 ± 0.55	3.24 ± 0.59	0.315
3	Alanine Transaminase (IU/L)	59.15 ± 32.03	56.73 ± 26.69	61.31 ± 36.10	0.271
4	Serum Total Cholesterol (mg/dl)	191.65 ± 46.07	193.25 ± 47.67	190.23 ± 44.74	0.616
5	Low density lipoprotein (mg/dl)	119.44 ± 41.26	120.96 ± 42.35	118.09 ± 40.38	0.593
6	High density lipoprotein (mg/dl)	36.31 ± 13.14	37.00 ± 12.91	35.69 ± 13.37	0.441
7	Triglycerides (mg/dl)	159.47 ± 96.89	162.08 ± 103.72	157.14 ± 90.74	0.695
8	Very-low density lipoprotein (mg/dl)	31.97 ± 19.44	32.64 ± 18.80	31.38 ± 20.05	0.620
9	Non-high density lipoprotein (mg/dl)	155.44 ± 43.02	156.55 ± 43.87	154.46 ± 42.39	0.710
10	Total cholesterol to HDL ratio	5.75 ± 2.50	5.57 ± 1.94	5.91 ± 2.92	0.311
11	LDL to HDL ratio	3.57 ± 1.84	3.49 ± 1.59	3.65 ± 2.05	0.515
12	Triglycerides to HDL ratio	5.25 ± 4.92	5.15 ± 4.89	5.33 ± 4.97	0.777

Around one-half of the study, the population had above-normal lipid markers, non-HDL most commonly deranged (73%), followed by HDL (71%). With respect to the ratios, LDL/HDL (73%) followed by TG/HDL (65%) were most likely deranged. The mean values were compared amongst fatty liver versus non-fatty liver groups revealed high creatinine, ALT, HDL, TG and VLDL in the fatty liver group. At the same time, LDL/HDL ratio was higher in the non-fatty liver group, as shown in Table 3.

**Table 3:** Comparison of lipid and biochemical markers amongst the status of NAFLD. P-Value calculated by independent sample t-test (* denotes significant values). Abbreviations: ALT, alanine transaminase; LDL, low-density lipoprotein; HDL, high-density lipoprotein; VLDL, very-low density lipoprotein; non-HDL, non-high density lipoprotein.

#	Laboratory investigations	Above/below given value	Grouping variables		p-value
			Fatty Liver (n=159)	No fatty liver (n=79)	
1	Serum Creatinine (>3.0 mg/dl)	n=198 (83.2%)	5.49 ± 3.58	4.37 ± 2.83	0.009*
2	Serum Albumin (<3.5 g/dl)	n=140 (58.8%)	3.27 ± 0.54	3.29 ± 0.64	0.837
3	ALT (>45 IU/L)	n=161 (67.6%)	65.33 ± 33.64	46.73 ± 24.32	<0.001*
4	Total Cholesterol (>200 mg/dl)	n=103 (43.3%)	193.93 ± 44.33	187.06 ± 49.35	0.279
5	LDL (>130 mg/dl)	n=79 (33.2%)	117.88 ± 40.58	122.58 ± 42.68	0.410
6	HDL (<40 mg/dl)	n=169 (71.0%)	38.97 ± 13.57	30.94 ± 10.40	<0.001*
7	Triglycerides (>150 mg/dl)	n=100 (42.0%)	169.20 ± 105.80	139.88 ± 72.60	0.013*
8	VLDL (>30 mg/dl)	n=101 (42.4%)	34.44 ± 19.77	27.05 ± 17.89	0.004*
9	Non-HDL (>130 mg/dl)	n=174 (73.1%)	154.83 ± 41.93	156.68 ± 45.39	0.756
10	Total cholesterol to HDL ratio (>5)	n=132 (55.5%)	5.57 ± 2.61	6.12 ± 2.25	0.112
11	LDL to HDL ratio (>2.5)	n=174 (73.1%)	3.36 ± 1.81	4.00 ± 1.84	0.012*
12	Triglycerides to HDL ratio (>3)	n=156 (65.5%)	5.52 ± 5.67	4.69 ± 2.82	0.224

ALT (p= 0.001) and VLDL (p<0.001) were more frequently deranged in fatty liver, while HDL (p= 0.016) and LDL/HDL ratio (p<0.001) were more frequently deranged in non-fatty liver group. The correlation statistics were revealing an inverse correlation of creatinine and albumin, a direct correlation of ALT and creatinine, while NAFLD was found correlated with creatinine (p<0.001), ALT (P<0.001), HDL (p<0.001), VLDL (p<0.001) and inversely related to Total cholesterol/HDL (p= 0.018), and LDL/HDL ratio (p<0.001) as given in Table 4.

**Table 4:** Correlation of lipid and biochemical markers amongst the status of NAFLD. * *indicates P-value was calculated by chi-square test.* r = correlation coefficient (Both Pearson’s and spearman’s correlation were used appropriately). Abbreviations: NAFLD, non-alcoholic fatty liver disease; ALT, alanine transaminase; LDL, low-density lipoprotein; HDL, high-density lipoprotein; non-HDL, non-high density lipoprotein; VLDL, very-low density lipoprotein; TG, triglycerides, TC, total cholesterol.

#	Laboratory investigations	Frequency: n(%)			Correlation coefficients			
		NAFLD (n=159)	No NAFLD (n=79)	P-value	Creatinine	ALT	Albumin	NAFLD
1	Creatinine (>3.0 mg/dl)	135 (84.9%)	63 (79.7%)	0.316^*^	-	r = 0.141, P=0.030	r = -0.273, P<0.001	r = 0.243, P<0.001
2	Albumin (<3.5 g/dl)	93 (58.5%)	47 (59.5%)	0.882^*^	r = -0.273, P<0.001	r = -0.116, P=0.075	-	r = -0.046, P=0.482
3	ALT (>45 IU/L)	119 (74.8%)	42 (53.2%)	0.001^*^	r = 0.141, P=0.030	-	r = -0.116, P=0.075	r = 0.254, P<0.178
4	Total Cholesterol (>200 mg/dl)	72 (45.3%)	31 (39.2%)	0.376^*^	r = 0.052, P=0.421	r = 0.026, P=0.691	r = 0.256, P<0.001	r = 0.103, P=0.112
5	LDL (>130 mg/dl)	50 (31.4%)	29 (36.7%)	0.417^*^	r = 0.020, P=0.755	r = 0.155, P=0.017	r = 0.326, P<0.001	r = -0.031, P=0.631
6	HDL (<40 mg/dl)	105 (66.0%)	64 (81.0%)	0.016^*^	r = 0.069, P=0.291	r = 0.000, P=0.997	r = 0.187, P=0.004	r = 0.317, P<0.001
7	Triglycerides (>150 mg/dl)	73 (45.9%)	27 (34.2%)	0.084^*^	r = -0.072, P=0.272	r = -0.095, P=0.145	r = -0.021, P=0.746	r = 0.122, P=0.061
8	VLDL (>30 mg/dl)	81 (80.2%)	20 (19.8%)	<0.001^*^	r = 0.006, P=0.926	r = 0.003, P=0.958	r = -0.027, P=0.676	r = 0.226, P<0.001
9	Non-HDL (>130 mg/dl)	119 (74.8%)	55 (69.6%)	0.392^*^	r = 0.033, P=0.616	r = 0.024, P=0.708	r = 0.204, P=0.002	r = 0.034, P=0.603
10	TC to HDL ratio (>5)	83 (52.2%)	49 (62.0%)	0.151^*^	r = 0.040, P=0.538	r = 0.232, P<0.001	r = 0.006, P=0.930	r = -0.153, P=0.018
11	LDL to HDL ratio (>2.5)	103 (64.8%)	71 (89.9%)	<0.001^*^	r = 0.033, P=0.616	r = 0.314, P<0.001	r = 0.131, P=0.044	r = -0.220, P=0.001
12	TG to HDL ratio (>3)	99 (62.3%)	57 (72.2%)	0.131^*^	r = -0.029, P=0.658	r = 0.060, P=0.360	r = -0.077, P=0.240	r = -0.017, P=0.790

**Figure 1:**
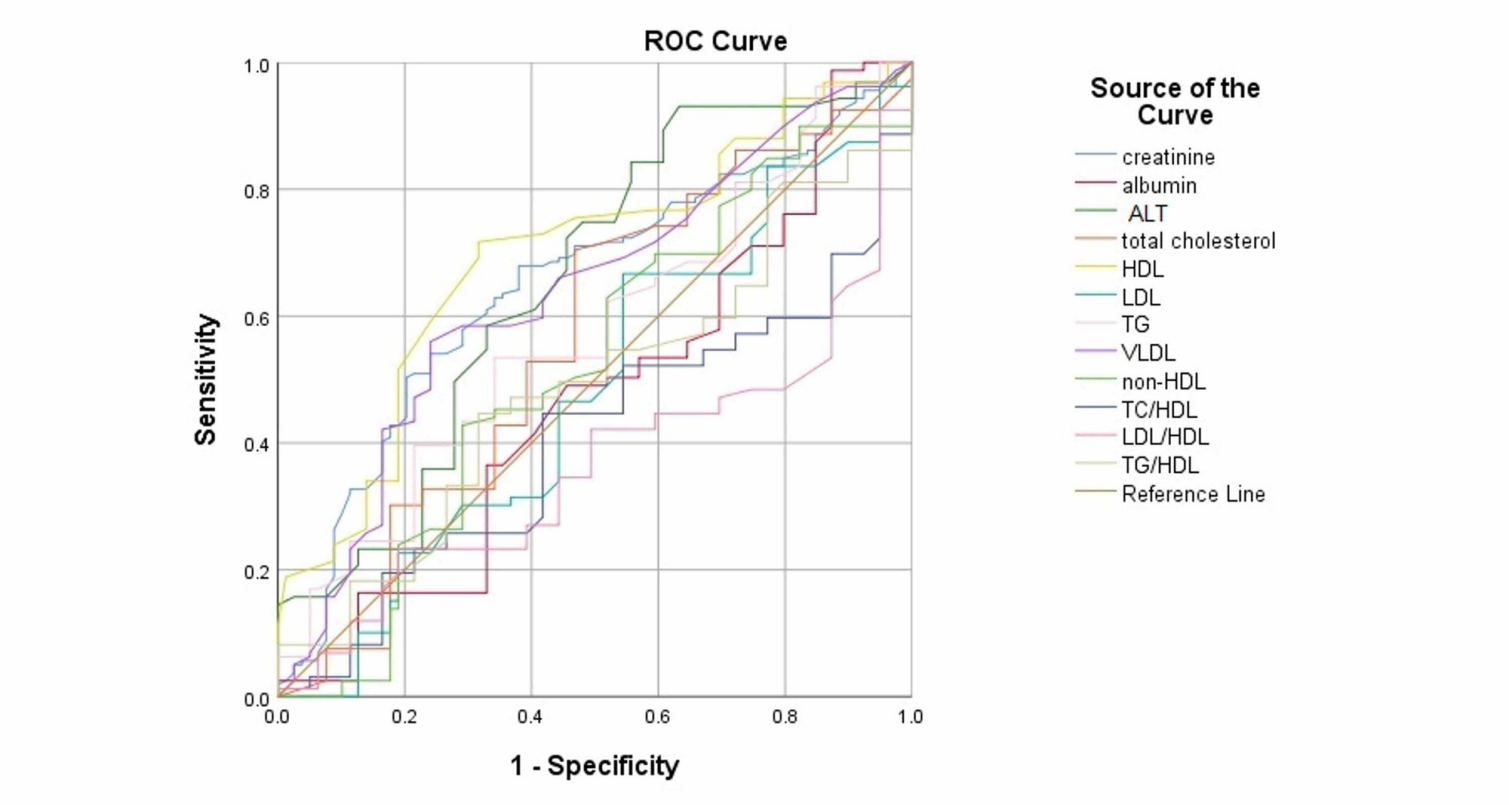
ROC curves for NAFLD group of CKD patients. Abbreviations: ROC, receiver operating characteristics; CKD, chronic kidney disease; NAFLD, non-alcoholic fatty liver disease; ALT, alanine transaminase; LDL, low-density lipoprotein; HDL, high-density lipoprotein; non-HDL, non-high density lipoprotein; VLDL, very-low density lipoprotein; TG, triglycerides, TC, total cholesterol.

**Figure 2:**
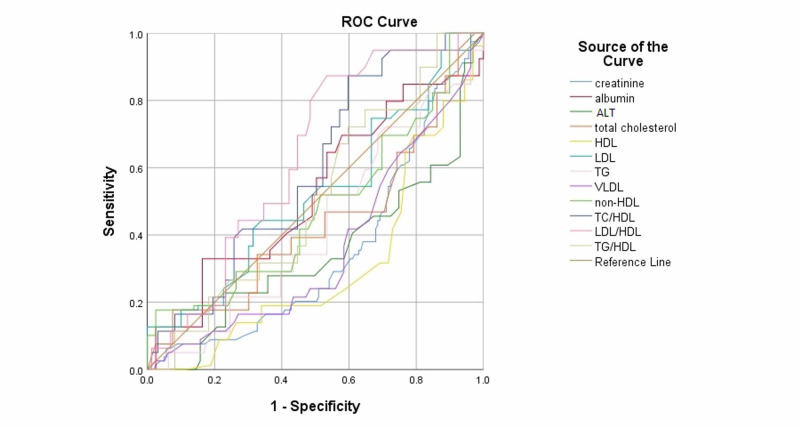
ROC curves for non-fatty liver groups of CKD patients. Abbreviations: ROC, receiver operating characteristics; CKD, chronic kidney disease; ALT, alanine transaminase; LDL, low-density lipoprotein; HDL, high-density lipoprotein; non-HDL, non-high density lipoprotein; VLDL, very-low density lipoprotein; TG, triglycerides, TC, total cholesterol.

Receiver operating characteristic analysis of the laboratory investigations showed creatinine (AUC: 0.649, p<0.001), ALT (AUC: 0.655, p<0.001), HDL (AUC: 0.694, p<0.001), VLDL (AUC: 0.637, p=0.001), Triglycerides (AUC: 0.575, p=0.061), and Total Cholesterol (AUC: 0.563, p <0.112) more increased in fatty liver group. While LDL/HDL ratio (AUC: 0.635, p<0.001) and TC/HDL ratio (AUC: 0.594, p=0.018) were found more increased in non-fatty liver group as shown in Table 5 as well as Figure 1 and Figure 2.

**Table 5:** ROC statistics of lipid and biochemical markers amongst the status of NAFLD. Abbreviations: ROC, receiver operating characteristics; AUC, area under the curve; S.E, standard error; 95% C.I, 95% confidence interval; ALT, alanine transaminase; LDL, low-density lipoprotein; HDL, high-density lipoprotein; non-HDL, non-high density lipoprotein; VLDL, very-low density lipoprotein; TG, triglycerides; TC, total cholesterol; NAFLD, non-alcoholic fatty liver disease.

#	Laboratory investigations	Characteristic Variable	AUC	S.E	95% C.I	p-value
1	Creatinine	NAFLD	0.649	0.037	0.576 – 0.722	<0.001
		No NAFLD	0.351		0.278 – 0.424	
2	Albumin	NAFLD	0.472	0.041	0.392 – 0.552	0.481
		No NAFLD	0.528		0.448 – 0.608	
3	Alanine Transaminase (ALT)	NAFLD	0.655	0.039	0.579 – 0.732	<0.001
		No NAFLD	0.345		0.268 – 0.421	
4	Total Cholesterol (TC)	NAFLD	0.563	0.041	0.483 – 0.644	0.112
		No NAFLD	0.437		0.356 – 0.517	
5	LDL	NAFLD	0.481	0.041	0.401 – 0.561	0.631
		No NAFLD	0.519		0.439 – 0.599	
6	HDL	NAFLD	0.694	0.036	0.624 – 0.764	<0.001
		No NAFLD	0.306		0.236 – 0.376	
7	Triglycerides (TG)	NAFLD	0.575	0.039	0.499 – 0.650	0.061
		No NAFLD	0.425		0.350 – 0.501	
8	VLDL	NAFLD	0.637	0.038	0.563 – 0.712	0.001
		No NAFLD	0.363		0.288 – 0.437	
9	Non-HDL	NAFLD	0.521	0.041	0.440 – 0.602	0.602
		No NAFLD	0.479		0.398 – 0.560	
10	TC/HDL ratio	NAFLD	0.406	0.037	0.333 – 0.479	0.018
		No NAFLD	0.594		0.521 – 0.667	
11	LDL/HDL ratio	NAFLD	0.365	0.036	0.294 – 0.436	0.001
		No NAFLD	0.635		0.564 – 0.706	
12	TG/HDL ratio	NAFLD	0.489	0.038	0.414 – 0.565	0.790
		No NAFLD	0.511		0.435 – 0.586	

## Discussion

A rise in plasma triglycerides, LDL, and VLDL, along with low HDL cholesterol is associated with chronic kidney disease [19]. The formation of atherosclerotic plaques in CKD due to mechanisms involving widespread inflammation, oxidative stress, and deranged lipid profile, most prominently HDL deficiency, plays a significant role in the increased risk of complications including cardiovascular diseases [20,21]. Thus, using lipid-lowering therapy in these patients is beneficial [22].

Non-alcoholic liver disease (NAFLD) is becoming increasingly more common with the rise in world obesity rates, and its underlying mechanisms indicate that it has a multisystem impact most prominently being associated with CKD, type 2 diabetes mellitus, cardiovascular disease among others [23]. Underlying risk factors such as obesity, dyslipidemia, hypertension, and insulin resistance in CKD and NAFLD are cross-linked and may explain the association between the two disease processes [24]. In a meta-analysis study conducted regarding the association of NAFLD with CKD, it was found that there was a link between the risk and severity of CKD and the presence and severity of NAFLD and emphasized the importance of implementing plans to prevent renal disease in this subset of patients [25]. A study conducted in India, focused on fasting lipid profile among CKD patients and concluded increased VLDL and TG and decreased HDL in hemodialysis patients as compared to those not on hemodialysis [3]. This finding was in contrast to our study, where we did not find any discrepancy of lipid markers in both the groups. Another study gave similar findings to our study by stating no difference in patients on hemodialysis; however, patients on peritoneal dialysis do exhibit dyslipidemia [5].

The comparative study had a mean age much higher in the hemodialysis group, as opposed to our study having no difference of age [3]. The fasting lipid profile levels in this study shown mean total cholesterol almost similar in hemodialysis (195) vs non-hemodialysis group (194), HDL (39 vs 37) and LDL (153 vs 149) slightly lower in the hemodialysis group, TG (187 vs 223) and VLDL (23 vs 34) significantly increased in hemodialysis group [3]. While in our study, total cholesterol (193 vs 190), LDL (120 vs 118), HDL (37 vs 35), TG (162 vs 157), VLDL (32 vs 31), and non-HDL (156 vs 154), all were comparable in both groups. Another study described HDL cholesterol deficiency and dysfunction, causing pro-inflammatory effects and has a role in the progression of CKD [21]. In our study, we found significantly decreased HDL in CKD patients; however, those with evidence of fatty liver disease had higher HDL. Despite higher HDL in the fatty liver group, CKD was more pronounced as evident by a comparatively higher serum creatinine in this group as compared to the non-fatty liver group. Hence, it can be postulated that the fatty liver has a direct correlation with the progression of CKD, rather than the levels of lipid markers.

Another population-based study was conducted on lipid markers in advanced CKD and found comparable to our study results such as total cholesterol (200 vs 191), LDL (116 vs 119), HDL (49 vs 36), TG (150 vs 159), non-HDL (151 vs 155), and TG/HDL ratio (4.0 vs 5.25). This study had a comparatively increased HDL while our study had a higher TG/HDL ratio [26]. Another study signified the association of NAFLD with the progression of CKD through decreased eGFR, a finding similar to our study, although we did not measure eGFR, rather significantly increased creatinine was found in the NAFLD group [27]. Another aspect of our study showed hepatic enzyme derangement, which was more pronounced in the NAFLD group as compared to non-fatty liver CKD patients. A similar study conducted showed hepatic enzymes were found decreased in the hemodialysis group, as compared to the non-hemodialysis group, a finding similar to our study having SGPT (56 vs 61), found lower in the hemodialysis group although statistically insignificant [28]. Lastly, many new therapeutic advances were signified by another study amongst CKD patients with NAFLD [29].

There were a few limitations of our study; first of all, we relied upon radiological evidence (ultrasonography) to pick fatty liver disease rather than histopathological evidence. Secondly, the results of the observational study design should not be considered accurate; hence further large-scale studies should be undertaken to confirm the findings of our study.

## Conclusions

The most exciting finding of our study was significantly higher HDL in fatty liver patients with CKD, while the non-fatty liver group of CKD patients showed decreased HDL levels. Hence, it can be concluded that the treatment options should not be targeted to improve HDL in CKD patients having fatty liver disease rather other groups of lipid-lowering drugs targeting triglycerides and total cholesterol synthesis discussed in the introduction should be utilized. Since it is a well-known fact that CKD itself causes lipid markers derangement without underlying NAFLD, hence the management options must be explored in those individuals with elevated fasting lipid profile focusing on improving HDL as identified by our study results along with other markers. While further large scale studies should take place in order to explore common therapeutics to deal with these two interlinked pathogenic entities.
